# An Emerging Foodborne Pathogen Spotlight: A Bibliometric Analysis and Scholarly Review of *Escherichia coli* O157 Research

**DOI:** 10.3390/antibiotics13010060

**Published:** 2024-01-08

**Authors:** Himanshu Jangid, Deepak Kumar, Gaurav Kumar, Raj Kumar, Narsimha Mamidi

**Affiliations:** 1 Department of Microbiology, School of Bioengineering and Biosciences, Lovely Professional University, Phagwara 144411, Punjab, India; himanshu.12116762@lpu.in; 2 Department of Chemistry, School of Chemical Engineering and Physical Sciences, Lovely Professional University, Phagwara 144411, Punjab, India; deepak.23418@lpu.co.in; 3 Department of Pharmaceutical Sciences, University of Nebraska Medical Center, Omaha, NE 68105, USA; 4 Wisconsin Center for NanoBioSystems, School of Pharmacy, University of Wisconsin-Madison, Madison, WI 53705, USA

**Keywords:** foodborne pathogens, food safety, emerging pathogens, *Escherichia coli* O157, bibliometric analysis

## Abstract

Foodborne infections pose a substantial global threat, causing an estimated 600 million illnesses and resulting in approximately 420,000 deaths annually. Among the diverse array of pathogens implicated in these infections, *Escherichia coli (E. coli*), specifically the O157 strain (*E. coli* O157), emerges as a prominent pathogen associated with severe outbreaks. This study employs a comprehensive bibliometric analysis and scholarly review focused on *E. coli* O157 research. The bibliometric analysis highlights the significant role played by the United States in the *E. coli* O157 research domain. Further exploration underscores the noteworthy contributions of the researcher Doyle MP, whose body of work, consisting of 84 documents and an impressive H-Index of 49, reflects their substantial impact in the field. Recent research trends indicate a discernible shift towards innovative detection methods, exemplified by the adoption of CRISPR-CAS and Loop-Mediated Isothermal Amplification. Moreover, high-throughput whole-genome sequencing techniques are gaining prominence for the expeditious analysis of pathogenic *E. coli* strains. Scientists are increasingly exploring antimicrobial agents, including phage therapy, to address the challenges posed by antibiotic-resistant *E. coli* strains, thereby addressing critical concerns related to multi-drug resistance. This comprehensive analysis provides vital insights into the dynamic landscape of *E. coli* O157 research. It serves as a valuable resource for researchers, policymakers, and healthcare professionals dedicated to mitigating *E. coli* O157 outbreaks and advancing global public health strategies.

## 1. Introduction

Foodborne infections persist as a considerable global health menace. According to data from the World Health Organization (WHO), nearly 600 million individuals suffer from illnesses resulting from the consumption of contaminated food each year, resulting in an estimated 420,000 deaths [1]. These infections not only pose immediate health hazards but also generate substantial economic consequences, manifested in escalating healthcare costs and diminished workforce productivity. Among the myriad of pathogens linked to foodborne diseases, *E. coli* occupies a distinctive position. This bacterium displays a captivating dual nature, functioning both as a benign inhabitant of the gut and a pernicious pathogen, rendering it a pivotal focus of scientific inquiry [2]. 

*E. coli* encompasses a diverse array of strains, with several exhibiting benign attributes essential to physiological processes such as vitamin K synthesis and the prevention of detrimental bacterial colonization. Notably, the O157 strain has drawn attention due to its association with severe foodborne outbreaks. Investigative studies have illuminated the multifaceted adaptive stress response of *E. coli* O157 when introduced into the human food chain, underscoring its distinctive survival characteristics that significantly influence its epidemiology and ecology. The consumption of the *E. coli* O157 strain can precipitate a spectrum of gastrointestinal complications. The particularly infamous subtype, *E. coli* O157:H7, is distinguished by its Shinga toxin production, which disrupts protein synthesis in human cells, ultimately culminating in cellular demise. This pathological process can manifest in a spectrum of symptoms ranging from bloody diarrhea to life-threatening conditions such as hemolytic uremic syndrome (HUS) [3].

Various research endeavors are underway to comprehensively elucidate the epidemiology, pathogenesis, transmission dynamics, and preventive strategies associated with *E. coli* O157. Concurrently, investigations into the role of external factors in the dissemination of *E. coli* O157 are being conducted. Notably, a study in Ohio, USA, explored the potential involvement of European starlings in the transmission of *E. coli* O157 among dairy farms, emphasizing the intricate web of transmission routes [4]. Similarly, a study in Syria utilized Random Amplified Polymorphic DNA (RAPD) markers to assess the genetic diversity of *E. coli* O157 in leafy greens irrigated by the Aleppo River, underscoring the significance of water sources in transmission dynamics [5]. Innovative approaches, such as Multiplex-PCR, are being employed for the rapid detection and comprehension of *E. coli* O157, as demonstrated in a study focusing on poultry, which simultaneously identified *Salmonella enteritidis*, *Shigella flexneri*, and *E. coli* O157: H7, emphasizing the importance of poultry as a potential source [6,7]. *E. coli* O157, particularly the H7 subtype, is predominantly identified in livestock, with cattle serving as significant carriers. This association underscores the frequent outbreaks associated with the consumption of raw or undercooked beef products, including ground beef. However, the spectrum of contamination extends beyond livestock to encompass fresh produce, unpasteurized dairy, and water sources. As a preventive measure, a comprehensive approach is imperative, integrating stringent agricultural and manufacturing practices, adherence to proper cooking temperatures, and public education on safe food handling [8].

In response to the escalating antibiotic resistance observed among various *E. coli* strains, the scientific community is actively exploring alternative therapeutic strategies [9,10]. Probiotics, such as *Lactobacillus rhamnosus*, have emerged as potential candidates offering protection against these pathogenic strains [11]. Simultaneously, nanotechnology is gaining prominence in the battle against bacterial infections. Nanoparticles, characterized by their minute size and expansive surface area, exhibit an enhanced interaction with microbial cells, augmenting their antimicrobial efficacy. Noteworthy examples include chitosan nanoparticles [12,13,14], especially when fortified with antimicrobial peptides, and metal oxide nanoparticles such as zinc oxide nanoparticles [NO_PRINTED_FORM], which are being investigated for their potent antibacterial properties against *E. coli* O157:H7 [15]. Additionally, lipid-based nanoparticles, known for their biocompatibility, are being tailored for targeted antibiotic therapy to mitigate the risk of resistance development [16].

In the face of the enduring global challenge posed by foodborne infections, gaining a nuanced understanding of pathogens such as *E. coli* O157 assumes paramount significance. The dual nature of *E. coli*, marked by its extensive strain diversity and the profound implications of its pathogenic variants, necessitates comprehensive research endeavors. Leveraging tools such as bibliometric analysis and innovative methodologies, the scientific community is dedicated to unraveling the epidemiology, transmission dynamics, and potential interventions associated with *E. coli* O157.

Bibliometric analysis, utilizing statistical and visualization tools, plays a pivotal role in decoding the extensive literature concerning this pathogen. Notably, researchers employing bibliometric analysis have successfully identified the key authors, tracked the evolution of research themes, pinpointed seminal papers, and elucidated research gaps [17]. In this paper, the amalgamation of bibliometric analysis and scholarly reviews aims to furnish a holistic perspective on *E. coli* O157 research, thereby bridging knowledge gaps and illuminating avenues for future exploration in alignment with the overarching objectives of this study.

## 2. Literature Review

### 2.1. Early Discoveries and Their Implications

Foundational research on *E. coli* O157 has paved the way for a deeper understanding of this pathogenic strain and its implications for public health. One of the seminal studies in this field was conducted by Morgan in their paper titled “Hemorrhagic Colitis Associated with a Rare *E. coli* Serotype”, published in 1988 [18]. This groundbreaking work identified *E. coli* O157 as the causative agent of a severe form of foodborne illness characterized by bloody diarrhea. Morgan and colleagues’ research not only established the association between *E. coli* O157 and hemorrhagic colitis but also highlighted the significance of this strain as a public health concern. Their findings underscored the need for enhanced surveillance and research efforts to understand the pathogenesis and epidemiology of *E. coli* O157 infections. Furthermore, this early research laid the foundation for subsequent investigations into the mechanisms of *E. coli* O157 virulence, its reservoirs in the environment, and strategies for preventing and managing outbreaks. The implications of this foundational work have reverberated through the years, influencing public health policies and interventions aimed at mitigating the risks associated with *E. coli* O157 infections.

### 2.2. Epidemiological Shifts

*E. coli* O157, commonly referred to as *E. coli* O157, is a significant public health concern due to its association with foodborne disease outbreaks. *E. coli* O157 is responsible for approximately 63,000 cases of hemorrhagic colitis annually in the United States. A comprehensive analysis of databases and studies from 10 out of 14 World Health Organization subregions revealed a global incidence of *E. coli*, totaling 2.8 million cases each year [19]. This pathogenic bacterium is often linked to the consumption of undercooked foods, particularly those contaminated with cattle manure harboring the bacterium. The epidemiological landscape of *E. coli* O157 is complex, with various factors influencing its prevalence and virulence. *E. coli* O157 primarily resides in cattle, especially those raised for beef, colonizing their intestines without noticeable symptoms. However, the bacteria from their fecal matter can contaminate food and water sources, causing human outbreaks. Understanding how *E. coli* O157 colonizes cattle is crucial for effective mitigation. Recent studies have highlighted gaps in knowledge regarding factors regulating its colonization, interactions with the host’s microbiota, and the host’s immune responses. Disturbances like stress during cattle production likely contribute to the bacterium’s survival and proliferation in the intestine [20]. Additionally, research has raised concerns about laboratory-acquired infections. A notable case involved a researcher contracting a severe *E. coli* O157 infection from nalidixic acid-resistant strains used in the lab, highlighting the bacterium’s virulence and emphasizing the need for strict safety protocols. The study also revealed that certain antibiotics, when given in sublethal doses, could enhance toxin expression, suggesting the potential exacerbation of infection severity with antibiotic treatment [21]. To gain insights into the epidemiological shifts and genetic aspects of *E. coli* O157, researchers have utilized murine models. These controlled environments enable the study of intestinal colonization, host responses, and the impact of host physiological status and microbiota on colonization and disease [20]. Such research offers a valuable understanding of host–pathogen–microbiota interactions, potentially leading to rational mitigation strategies for *E. coli* O157 in cattle.

### 2.3. Pathogenesis of E. coli O157

**Figure 1:**
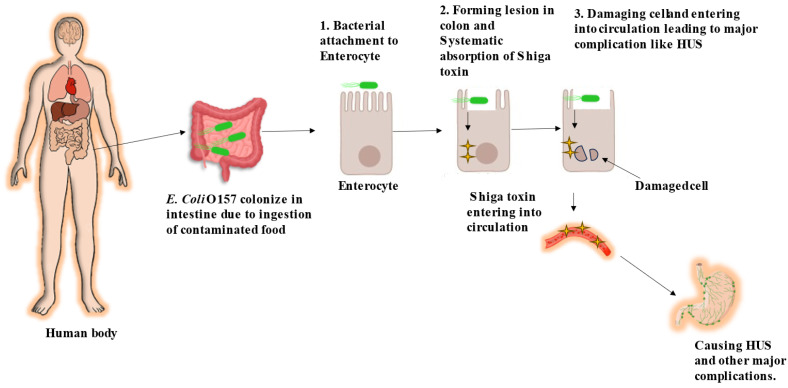
Pathogenesis of *E. coli* O157 in human [19].

*E. coli* O157 stands out as a highly potent strain of enterohemorrhagic *E. coli* (EHEC). Primarily, cattle are a major natural reservoir of the *E. coli* O157 strain [22]. Furthermore, these cattle transmit *E. coli* O157 either through farm or food products to humans, where they survive in the gastrointestinal tract and cause infection. Its virulence lies in its ability to inflict extensive harm within the host, leading to various conditions, from mild gastroenteritis to severe hemorrhagic colitis and life-threatening hemolytic uremic syndrome (HUS). The pathogenicity of *E. coli* O157:H7 involves an intricate series of steps, incorporating bacterial adherence, toxin production, and strategies to evade the immune system [23]. Figure 1 visually represents the pathogenesis of *E. coli* O157 infection in humans. 

**Figure 2:**
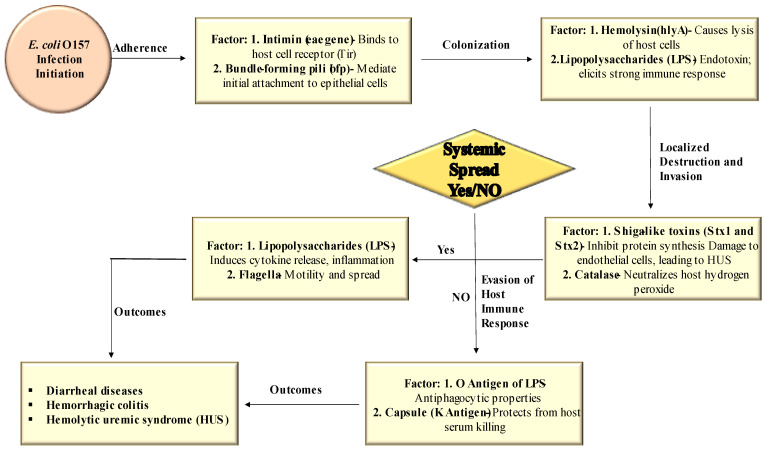
Role of different virulence factors at each stage of *E. coli* O157 infection.

Various virulence factors play a role in the pathogenesis of *E. coli* O157 infection. Figure 2 visually represents the different virulence factors involved in each stage of *E. coli* O157 infection.

### 2.4. Antimicrobial Strategies against Escherichia coli O157: Traditional and Emerging Approaches

*Escherichia coli* O157 has become a notable public health issue, particularly in the realm of foodborne illnesses. Dealing with infections caused by this pathogen has proven to be difficult due to its virulence and escalating resistance to conventional antimicrobials [24]. This section concentrates on a variety of antimicrobial approaches employed against *E. coli* O157, encompassing both established treatments and inventive methods that have arisen in response to concerns about antibiotic resistance.

In the management of *E. coli* O157 infections, traditional treatment approaches have heavily relied on antibiotics such as ciprofloxacin, tetracycline, sulfamethoxazole, and penicillin [25]. However, the escalating resistance to these antibiotics has raised significant concerns [26]. Notably, *E. coli* O157 has demonstrated substantial resistance to antibiotics like tetracycline, sulfamethoxazole, and erythromycin, particularly in strains isolated from various sources, including pigs, cattle, and humans [27]. This increasing resistance pattern complicates existing treatment strategies and emphasizes the need for a more in-depth understanding of the mechanisms of antibiotics and the dynamics of resistance development. In response to the challenges posed by antibiotic resistance, research efforts have shifted towards alternative antimicrobial strategies. One promising avenue involves the exploration of natural compounds and probiotics [28]. For example, a specific combination of natural antimicrobials has exhibited effectiveness against *E. coli* O157 in both in vitro studies and animal models, suggesting its potential use in controlling this pathogen within the animal gut. Additionally, combinations of plant extracts and organic acids have been found to diminish the virulence of *E. coli* O157, presenting an innovative approach to managing this pathogen effectively [29].

Beyond natural compounds, the scientific community is also investigating the potential of bacteriophages in controlling *E. coli* O157:H7. These bacteriophages can be administered through various means, such as oral or rectal administration, to ruminants or through spraying or immersion treatments for fruits and vegetables. This versatility positions them as a significant tool in combating *E. coli* O157:H7 [30]. Moreover, mucoadhesive chitosan microparticles have been identified as potent bactericidal agents capable of disrupting cell membranes, making them a promising option for treating infections caused by *E. coli* O157:H7 [7,31] and highlighting their ongoing innovation in antimicrobial research.

The subsequent Table 1 provides a comprehensive overview of both the traditional and novel antimicrobials used against *E. coli* O157. It outlines their mechanisms of action, effectiveness, applications, and notes on resistance, offering a holistic perspective on current and prospective antimicrobial strategies. This comparison not only illuminates the changing landscape of antimicrobial resistance but also underscores the innovation in antimicrobial research, which is crucial for addressing the challenges posed by *E. coli* O157.

### 2.5. Innovations in Detection and Prevention

In recent times, significant progress has been made in enhancing the detection and prevention methods related to *E. coli* O157, leading to notable improvements in food safety and public health. The introduction of innovative technologies has transformed our capabilities in identifying and combating this bacterium effectively. These advancements include a wide range of techniques, from rapid molecular assays to sophisticated biosensors, all aimed at improving the sensitivity, specificity, and efficiency of *E. coli* O157 detection [52].

Within our extensive review, we compiled a table featuring the latest 20 state-of-the-art detection techniques (as mentioned in Table 2). Each method represents a distinct approach to diagnosing *E. coli* O157 infections, incorporating cutting-edge developments in molecular biology, immunological assays, nanotechnology, and other relevant fields. Our exploration of this diverse array of innovations aims to provide a comprehensive understanding of the current landscape of *E. coli* O157 detection strategies. This analysis sheds light on the progress achieved thus far and offers insights into potential directions for future research and development in this crucial area.

## 3. Methodology

### 3.1. Data Sources and Selection

Data were taken from the Scopus database on 20 October 2023. Scopus has the most extensive databases containing abstracts and citations from the peer-reviewed literature, spanning diverse subjects and offering a comprehensive overview of the research landscape. Utilizing Scopus provided several advantages for our study which included broad coverage as a vast array of journals across multiple disciplines were considered, ensuring a comprehensive perspective on *E. coli* O157 research, high-quality data as the Scopus database maintains fixed selection criteria indexing only reputable and impactful journals, interdisciplinary insights, and lastly, advanced search and analytical tools, which provide streamlining data retrieval and preliminary analysis. Furthermore, during data collection, we set certain exclusion criteria, like studies encompassing only peer-reviewed articles directly related to the topic. Articles in languages other than English, those lacking abstracts, and duplicates were meticulously excluded from our dataset [72].

### 3.2. Bibliometric Tools and Metrics

Bibliometric analysis offers a quantitative approach to understanding the patterns, networks, and impact within specific research areas. In our study on *E. coli* O157, we utilized various advanced bibliometric tools for a comprehensive analysis:

➢ VOSviewer: This tool facilitated the visualization and analysis of bibliometric networks, mapping co-authorship patterns and revealing collaborative clusters among researchers and institutions. Its keyword co-occurrence analysis unveiled central themes and emerging trends in the field [73].
➢ R Studio: We employed R Studio along with tailored bibliometric packages and scripts to conduct in-depth analyses, including co-citation assessments. Its flexibility and statistical capabilities enabled us to visualize complex bibliometric data effectively [74].
➢ Thematic Mapping: To visually represent the thematic evolution in *E. coli* O157 research, we employed thematic mapping techniques. This involved clustering-related keywords and topics to identify distinct thematic areas and tracing their emergence, convergence, and divergence over time. This mapping highlighted dominant research themes, emerging areas, and potential gaps in the literature [75].
  We focused on pivotal metrics for a comprehensive bibliometric assessment:
➢ Publication Count: This metric provided insights into research volume and growth, revealing periods of heightened activity [76].
➢ Citation Analysis: Examining citation patterns pinpointed influential papers, authors, and institutions, indicating the impact and recognition of specific works within the research community [77].
➢ Co-authorship Networks: These networks illuminated collaborations, showcasing influential research groups and their contributions, as well as the interconnectedness of the global research community [78].
➢ Keyword Analysis: By examining keyword frequency and co-occurrence, we traced the evolution of research themes, identifying dominant topics, emerging interests, and potential gaps [79].

Through these tools, metrics, and thematic mapping techniques, our bibliometric analysis aimed to provide a detailed, data-driven overview of the *E. coli* O157 research landscape, capturing its complexities and evolution.

## 4. Results and Discussion

**Figure 3:**
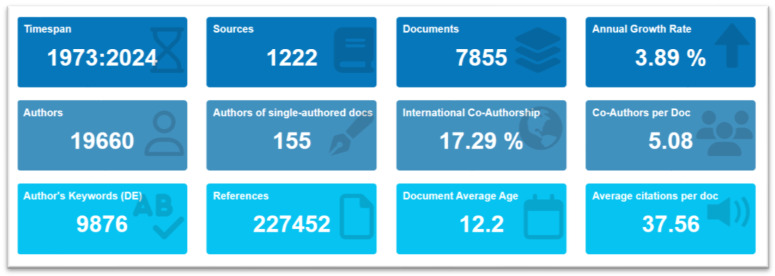
Visualization of collected data from Scopus database.

Data were collected from the Scopus database on 20 October 2023 using a specific search string: TITLE-ABS-KEY (“*E. coli* O157”) combined with filters to limit the results to the English language and document type “AR”. To ensure the integrity and quality of the data, an initial cleaning process was undertaken using Zotero Version 6.0.27, which effectively removed duplicate entries. Following this, a rigorous data processing phase was executed, wherein a total of 669 publications were excluded based on the following specific criteria: 250 review articles, 233 conference papers, 86 book chapters, 43 letters, 51 other types of articles, and 6 editorials. After these exclusions, a total of 7855 publications remained. These were then subjected to quantitative analysis using R Studio (Version R 3.3.0, 2023) with the bibliometrix R package. For visualization purposes, Vos Viewer Version 1.6.19 was employed. Any subsequent data preparation and adjustments were carried out using Microsoft Excel 2021 to ensure the data were primed for in-depth analysis. Figure 3 shows an overview of collected data from the Scopus database.

### 4.1. Evolution of Publications over Time

The research landscape on *E. coli* O157 has seen a dynamic evolution since its inception. The foundational paper in this domain was published in 1973 [80], bearing the title “Isolation of *E. coli* O157 from pigs with colibacillosis in Canada and the United States.” Following this groundbreaking work, a noticeable gap emerged with no publications from 1974 to 1981. A modest revival was observed between 1982 and 1989, with less than 10 papers. However, the subsequent years, particularly post-1989, marked a significant uptick in research interest, culminating in peak numbers of publications of 377 and 368 in 2015 and 2020, respectively. As of 2023, the momentum remains robust, with 230 papers already published.

This trend analysis provides valuable insights into the shifting priorities and focus of the scientific community. The hiatus posts that the 1973 publication might reflect the challenges faced in the nascent stages of research, potentially due to technological constraints, funding limitations, or the preliminary nature of the topic. The modest activity between 1982 and 1989 suggests a budding but cautious interest. The exponential growth post-1989, however, underscores the escalating significance of *E. coli* O157 in both clinical and research contexts.

**Figure 4:**
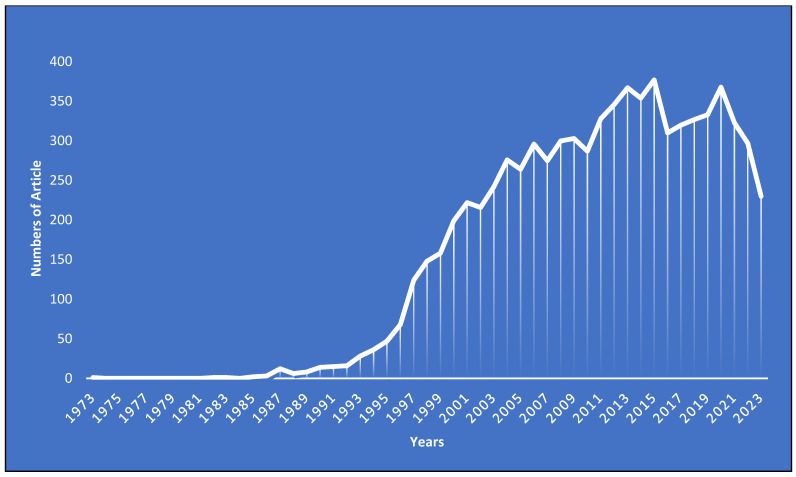
Yearly published research articles on *E. coli* 0157 as the research domain.

The peak years of 2015 and 2020 stand as a testament to the culmination of research endeavors and the global emphasis on understanding this bacterium. The sustained engagement in 2023 indicates not only the continued relevance but also hints at emerging challenges or innovations that keep researchers intrigued (please refer to Figure 4). In summary, the evolution of publication trends offers a comprehensive view of the historical progression of *E. coli* O157 research. It serves as both a reflection of past endeavors and a beacon, highlighting potential avenues and gaps for future exploration.

### 4.2. Publication Distribution Based on Region

**Figure 5:**
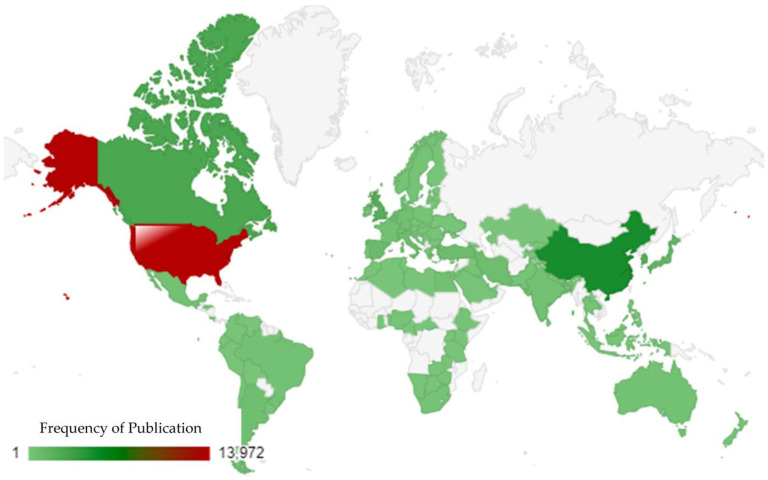
Visualization of region-based distribution of publications using a world map.

The regional distribution of research on *E. coli* O157 offers a compelling insight into global research dynamics [81]. The USA distinctly leads the forefront, followed by China, Canada, South Korea, and the UK. The USA’s dominance in this research domain might be attributed to its robust research infrastructure, substantial funding opportunities, and a long-standing tradition of scientific inquiry. With a frequency of 13,972, it significantly outpaces other nations in its contributions. China, holding the second position with a frequency of 5027, underscores its rapid ascent in the global research arena. This reflects China’s growing scientific capabilities and its emphasis on health-related research. Canada’s contribution, with a frequency of 2803, might be influenced by its proximity to the USA and shared concerns regarding public health. South Korea, with a frequency of 2240, showcases its commitment to scientific advancements in health and microbiology. The UK, with a rich history of scientific research, registers a frequency of 2161, emphasizing its continued role in global health research. In essence, this geographic distribution underscores the universal significance of research on *E. coli* O157 (as shown in Figure 5). While certain regions lead in terms of sheer volume, the collective effort from various countries highlights the global commitment to understanding this bacterium.

**Figure 6:**
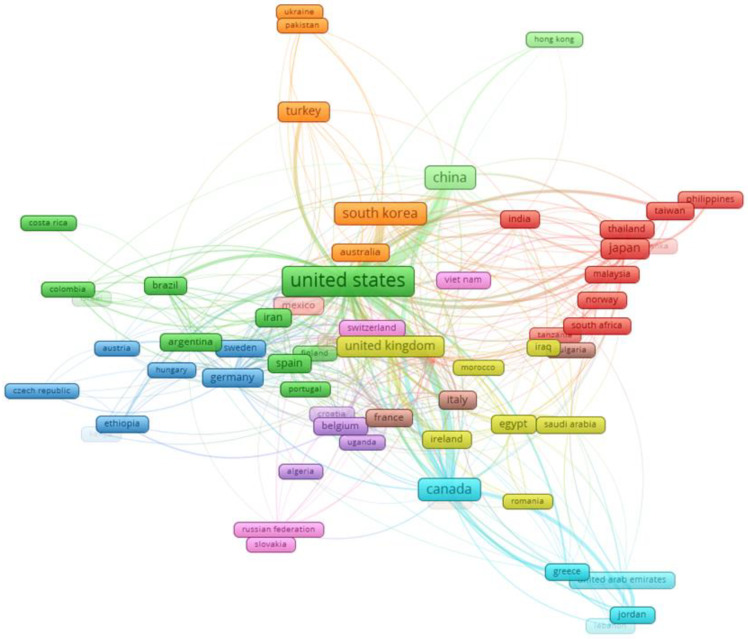
Visualization of country collaboration network using Vosviewer.

Additionally, Vosviewer was employed to visualize collaboration networks among countries, specifically concentrating on those nations with at least 5 documents out of the total 164 countries. Seventy-three countries met this criterion and were chosen for analysis, as depicted in Figure 6.

### 4.3. Co-Authorship Analysis

The utilization of bibliometric analysis to examine co-authorship patterns provides valuable insights into the collaborative dynamics inherent within the scientific community [82]. Our comprehensive data analysis encompassed a vast pool of 7456 authors who have made significant contributions to the field of *E. coli* O157 research. To facilitate a more detailed investigation, we applied the criterion of selecting authors with a substantial body of work, specifically those with more than five publications in this domain, ensuring that we focused on individuals with a considerable impact in the field.

Within this cohort of prolific authors, five individuals emerged as the foremost contributors to *E. coli* O157 research, each distinguished by their exceptional publication records. Notably, Doyle MP, affiliated with the Department of Food Microbiology and Toxicology and the Food Research Institute at the University of Wisconsin, Madison, USA, stands out prominently. Doyle’s remarkable portfolio comprises an impressive 84 documents and a substantial H-Index of 49, firmly establishing him as a preeminent figure in the realm of *E. coli* O157 research. Furthermore, an exploration of his collaborative network reveals a robust total link strength, indicative of the extensive connections he has forged within the research community. Table 2 below shows the top 10 most prolific authors in the field of the *E. coli* O157 research domain.

**Figure 7:**
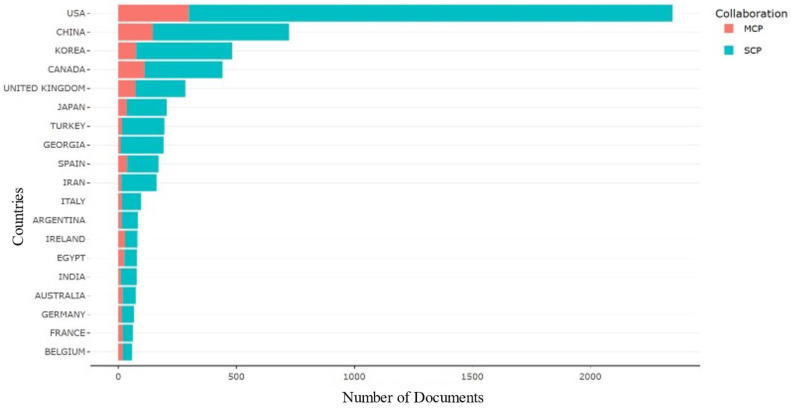
Countries collaboration based on MCP and SCP.

Examining collaboration patterns among countries using the Multiple Countries Publications (MCP) and Single Country Publications (SCP) metrics has yielded crucial information about international partnerships in *E. coli* O157 research [83]. The analysis reveals that the USA leads both single-country publications and extensive collaborations with multiple countries, indicating its prominent role in this research area. Following closely behind are China and Korea, emphasizing their active involvement in both single-country and collaborative publications within this domain, as shown in Figure 7. 

**Figure 8:**
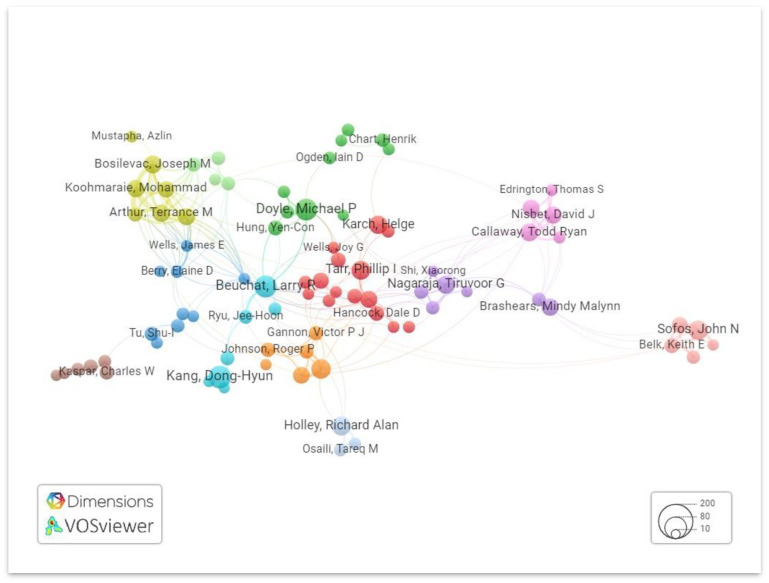
Network visualization of the collaboration network of authors. Different clusters are denoted by a different color.

Vosviewer is used to visualize the collaboration network of authors based on total linkage strength, as shown in Figure 8. Criteria were set to authors with a minimum of five research articles, which were selected for analysis out of 7456 authors.

**Figure 9:**
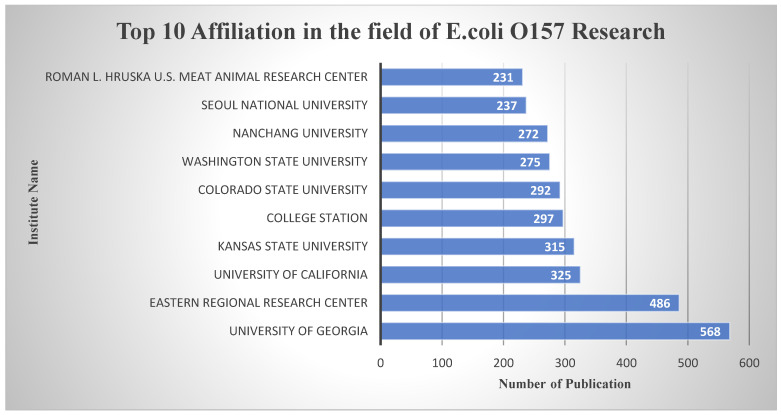
Top 10 affiliations in the field of *E. coli* O157 Research domain based on publication number.

Turning our attention to institutions, the University of Georgia and the Eastern Regional Research Centre have assumed leadership positions as the top institutional contributors in the domain of *E. coli* O157 research, as shown in Figure 9. The University of Georgia, renowned for its research excellence, has made a substantial impact with 568 published documents. Meanwhile, the Eastern Regional Research Centre has made significant strides with 486 publications, reflecting their dedication to advancing knowledge in this crucial area.

### 4.4. Keywords Analysis

Examining keywords in bibliometric research has proven to be a robust approach for revealing and comprehending the fundamental themes, trends, and intellectual structure within a particular research area [84]. In our investigation, we employed a dataset obtained from the Scopus database and conducted a comprehensive analysis of keyword co-occurrence.

Among the 28,269 identified keywords, 9899 were author keywords, while the rest were index keywords. We focused on author keywords that appeared more than five times, categorizing them as high-frequency keywords for our analysis. As a result, out of the 9899 author keywords, 704 met this criterion and were included in our study.

The most frequently occurring author keyword was “*Escherichia coli* O157:H7”, noted 1065 times, with a substantial total linkage strength of 2218. Following closely were the keywords “*Escherichia coli*” and “*Escherichia coli* O157,” appearing 328 and 213 times, respectively, with total linkage strengths of 761 and 435, as detailed in Table 3.

**Figure 10:**
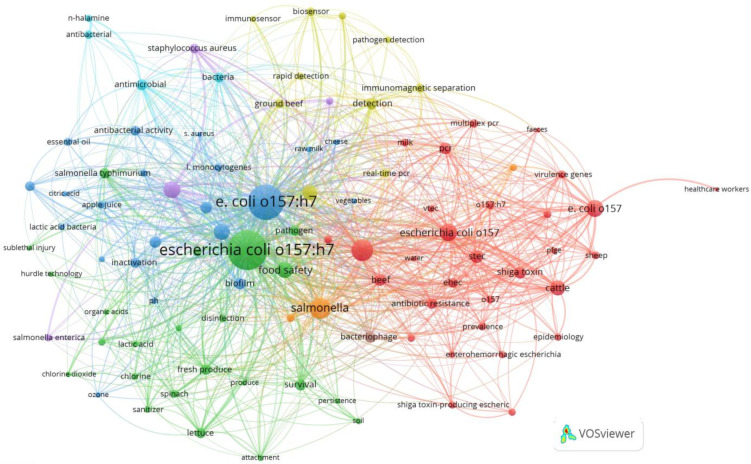
Keyword co-occurrence network visualization using Vosviewer. The size of the nodes signifies the importance or prominence of items in the dataset, with larger nodes indicating greater importance. The color of the nodes categorizes items based on characteristics, helping to identify clusters or groups within the network, with each color representing a different category or group.

**Figure 11:**
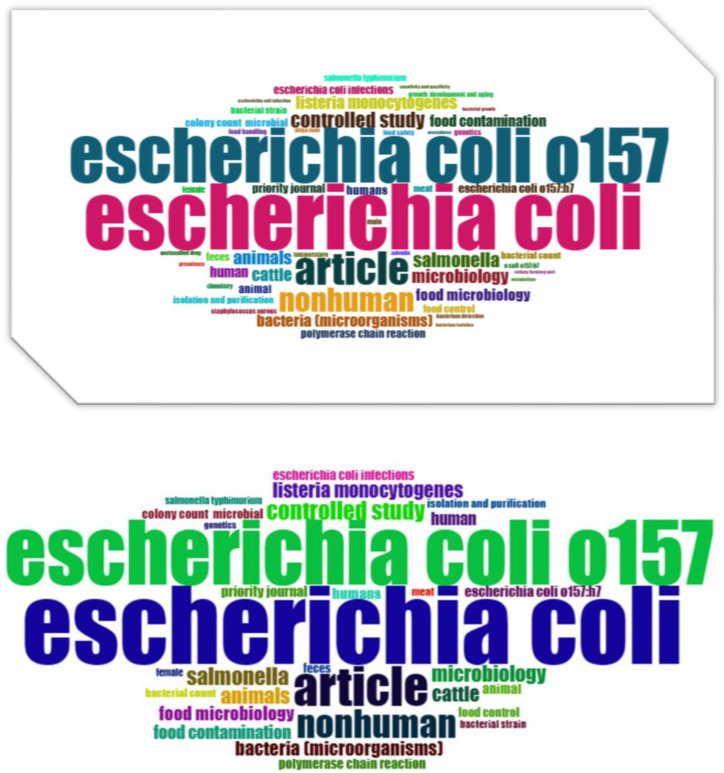
Word cloud prepared using R-studio. The size of word indicates the number of co- occurrences of the keyword. Text size in Figure 11 depicts the number of occurrences of the keyword, providing a visual representation of its frequency.

To visually depict the co-occurrence network of these keywords, we employed VOSviewer, as illustrated in Figure 10. Furthermore, Figure 11 displays a word cloud created using R Studio, emphasizing the most frequently appearing keywords based on their size.

**Figure 12:**
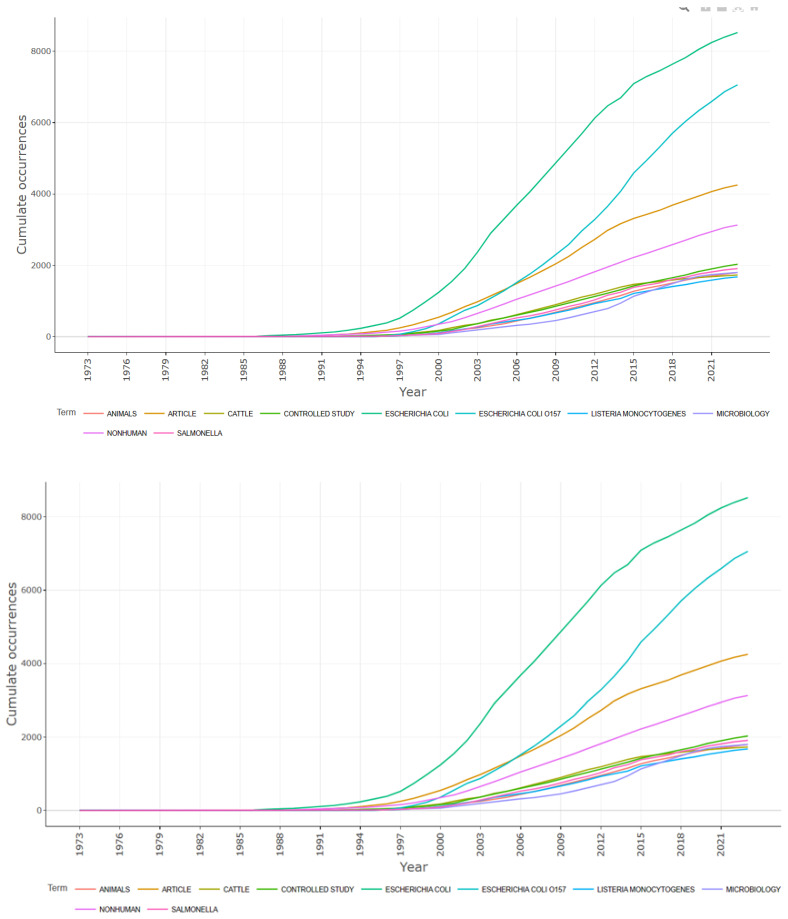
Word frequency over the time prepared using R-studio.

Additionally, utilizing the R-studio R package bibliometrix, we generated a word frequency graph based on the occurrence of keywords across different years. This graph highlights the evolving landscape of research within the *E. coli* O157 research domain over time, as shown in Figure 12.

**Figure 13:**
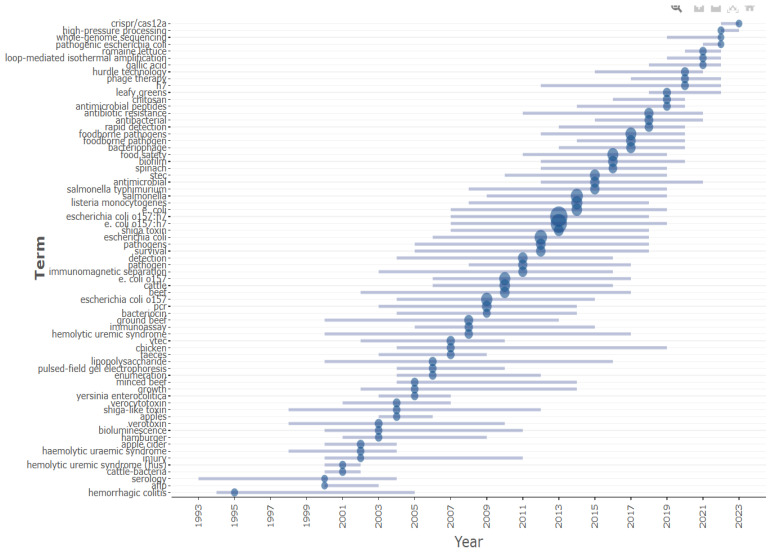

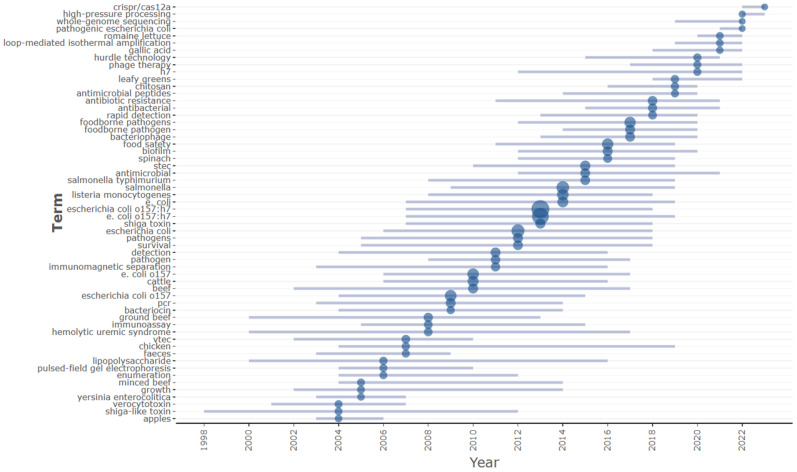
Keyword arrangement year wise; the line represents the corresponding year and the blue circle size indicates the number of occurrences. Trend topic analysis using R-studio.

Analyzing trending topics using the R-Studio Bibliometrix package has provided insights into emerging themes in *E. coli* O157 research based on keyword co-occurrence analysis, as shown in Figure 13. This analysis indicates a recent shift in focus within the last five years. There has been increased emphasis on the development of detection techniques, particularly those utilizing CRISPR-CAS and Loop-Mediated Isothermal Amplification methods. Additionally, there is a growing interest in advancing high-throughput whole-genome sequencing techniques for the rapid sequencing of pathogenic *E. coli* strains. The research spotlight has also turned towards the development of antimicrobial agents to combat antibiotic-resistant *E. coli* strains, including innovative therapies such as phage therapy, aiming to address the challenges posed by multi-drug resistance in *E. coli* strains.

### 4.5. Citation Analysis

This segment shows the substantial contributions made by prior research and scholarly works, which have greatly influenced and enhanced the content of our study [85]. The subsequent citation analysis presents a comprehensive compilation of references utilized throughout our research, spanning academic papers, books, articles, and other credible sources. These references form a robust basis for our exploration of the *E. coli* O157 research domain. Table 4 below lists the top 10 most-cited documents in the field of the *E. coli* O157 research domain.

After the most cited documents come the most impactful journals published; Table 5 below provides an overview of the most cited journals, accompanied by their H, G, and M indexes, offering a complete perspective on the highly influential journals within this research area.

**Figure 14:**
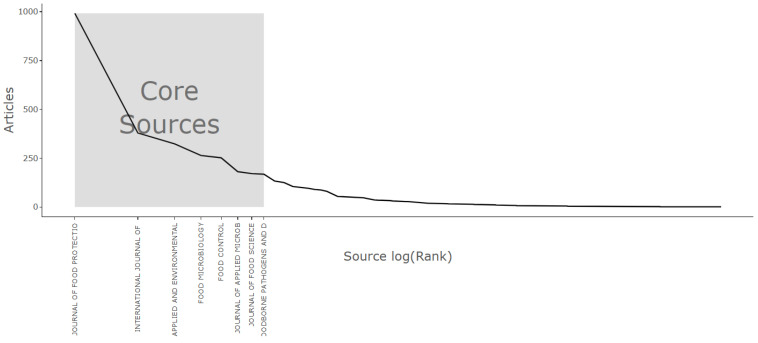
Core source analysis based on Bradford’s Law.

Bradford’s Law, a pivotal notion in bibliometric research, provides valuable perspectives on the unequal dispersion of information and scholarly publications within particular academic domains. This concept was formulated during the 1930s by Samuel C. Bradford, a British librarian. It suggests that within any given field of study, the scholarly literature frequently exhibits a discernible pattern: a nucleus of exceptionally prolific journals or sources, succeeded by a region where returns on information tend to diminish, and ultimately, an area characterized by dispersed or less central sources. Bradford’s Law has played a crucial role in enhancing our comprehension of the clustering of scientific knowledge and assisting researchers in refining their approaches to searches made in the literature [96]. Using this approach, Figure 14 shows core sources based on the number of articles using Bradford’s Law.

Further Table 6 shows most cited journal in the research domain of *E. coli* O157. A deeper analysis of citations highlights the most frequently cited countries, with the USA leading significantly with a total citation count of 105,195 and an average of 44.80 citations per article. Table 7 below illustrates this dominance, showing the USA as the top country in terms of citation numbers. China, listed second, lags far behind with 17,814 citations, indicating a substantial difference compared to the USA’s citation count.

### 4.6. Three-Field Plot

**Figure 15:**
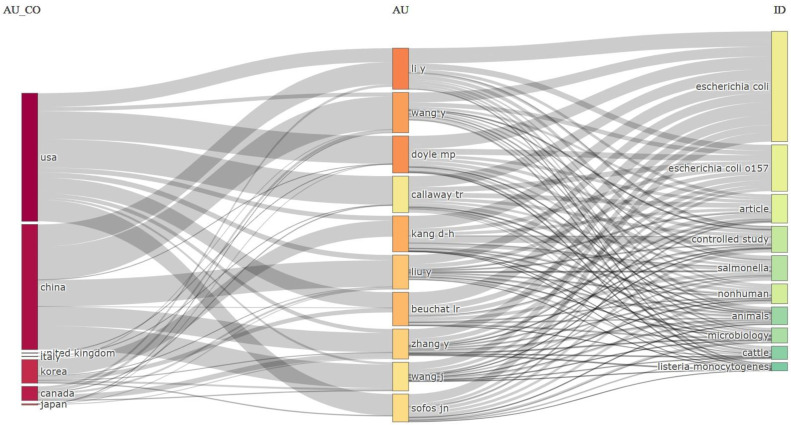
Three-field plots (Sankey diagram) based on the author’s country (**Left**), author (**Middle**), and keyword (**Right**).

This visual representation is a three-field plot, commonly known as a Sankey diagram or Alluvial diagram, depicting relationships between various data dimensions [97]. Specifically, it illustrates the interconnections among authors’ countries (AU_CO), individual authors (AU), and the topics or keywords of their research (ID) in the realm of *E. coli* O157 research, as shown in Figure 15.

In the leftmost column (AU_CO), different countries are represented by dark bars indicating their respective contributions to the research domain. The United States (USA) exhibits the most substantial presence, followed by China, the United Kingdom, Korea, Canada, and Japan. This suggests that these nations have active research communities dedicated to *E. coli* O157.

The middle column (AU) displays individual authors, with color-coding that likely signifies publication count or contribution weight, although this aspect is not clarified in the image. The width of the lines connecting authors to their countries and research topics reflects the volume of work or the strength of their association. Authors such as “Li Y” and “Wang Y” have extensive collaborations connections, suggesting a significant number of publications or a strong association with the listed topics.

The rightmost column (ID) showcases topics, identifiers, or keywords related to research publications. “*Escherichia coli*” and “*Escherichia coli* O157” emerge as the most prevalent topics, indicating a substantial focus on these bacteria. Additional linked topics encompass the following: “article”, “controlled study”, “*salmonella*”, “nonhuman”, “animals”, “microbiology”, “cattle”, and “*listeria monocytogenes*”. This diversity signifies varied yet interconnected areas of interest within *E. coli* O157 research. The inclusion of keywords like “cattle” and “*listeria monocytogenes*” implies research spanning beyond *E. coli* O157 to encompass related topics in food safety and zoonotic diseases. In summary, this three-field plot provides a visual summary of the *E. coli* O157 research landscape, showcasing active countries, influential authors, and frequently studied topics. It offers insights into the collaborative network and thematic focus within the research community studying *E. coli* O157.

### 4.7. Thematic Mapping and Factorial Analysis

**Figure 16:**
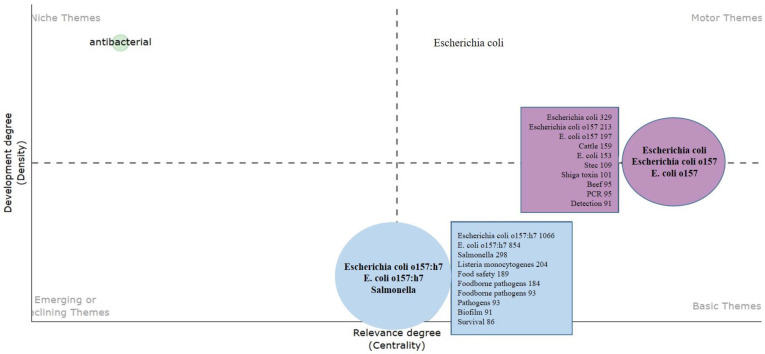
Theme map based on the *E. coli* O157 research domain.

A thematic map is commonly employed in bibliometric studies to visually depict the structure of a research field [98]. Here, it focuses on research related to *E. coli* O157, a specific strain of the *E. coli* bacterium known for causing foodborne illnesses, as shown in Figure 16. The map is segmented into four quadrants based on two axes: the vertical axis representing the development degree or density (indicating internal connections between themes), and the horizontal axis representing the relevance degree or centrality (indicating interactions with other themes). The following provides a breakdown of each quadrant:

1. Motor Themes (upper right): These themes are both well-developed and central, signifying their maturity and strong interconnections with other research topics. The prominent bubble in this quadrant is labeled “*Escherichia coli*”, “*E. coli* O157”, and “*E. coli* O157”, highlighting it as a core topic in the field with extensive research. Smaller bubbles also focus on *E. coli* O157 and related subtopics such as “cattle”, which is significant due to cattle being a primary reservoir for this bacterium.
2. Niche Themes (upper left): These themes are less developed but still represent niche areas of emerging or specialized interest. The single theme “antibacterial” suggests a concentration on treatments or resistance issues related to *E. coli* O157.
3. Basic Themes (lower right): These themes are well-developed but less central, foundational to the field, but not at the forefront of current research. This quadrant includes themes like “food safety”, indicating substantial research but is potentially less connected to other themes.
4. Emerging or Declining Themes (lower left): These themes have lower density and centrality, indicating that they are either new areas of interest yet to be fully explored (“emerging”) or areas losing focus within the research community (“declining”). Themes like “*E. coli* O157:H7” and “*salmonella*” fall into this category, suggesting some focus on these pathogens, though not necessarily as primary subjects or possibly newly emerging subjects within the field.

Each bubble within the quadrant represents a cluster of themes, with its size denoting the volume of research or attention given to that topic. The numbers alongside each term could indicate the number of publications or the weight of the theme in the analysis.

Researchers can use this map to identify active, emerging, and underexplored areas in *E. coli* O157 research. Funding agencies can utilize it to allocate resources effectively, while new researchers can identify gaps in the existing literature.

**Figure 17:**
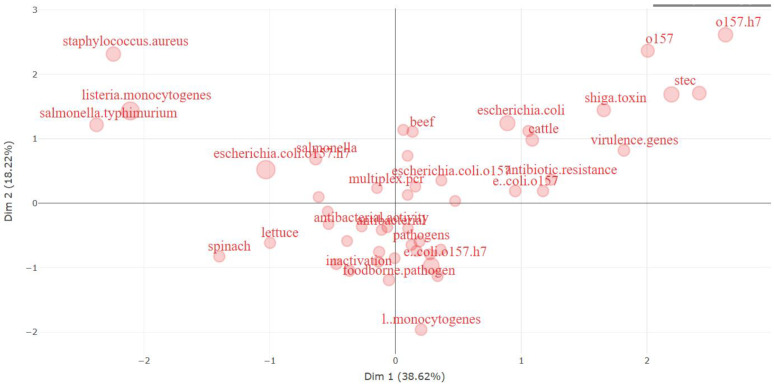
Visual representation of multiple correspondence analysis (MCA) based on the *E. coli* O157 research domain.

Figure 17 below is a visual representation resulting from a multiple correspondence analysis (MCA), portraying research topics associated with *E. coli* O157 in a spatial arrangement. MCA, a statistical method, aims to position each object in a multidimensional space, preserving the distances between objects as accurately as possible. In this context, each point on the plot represents a specific research topic or keyword, and their proximity signifies their closeness in the realm of research in the literature [99].

Breaking down the image:

➢ Dimension 1 (horizontal axis, explaining 38.62% of the variance): This dimension likely signifies a continuum in research topics, ranging from general *E. coli*-related subjects on the left to more specific subtopics or issues concerning *E. coli* on the right. Topics of higher specificity or those currently prominent in research tend to cluster towards the right end of this axis.
➢ Dimension 2 (vertical axis, explaining 13.29% of the variance): This dimension captures another aspect of variation among research topics, potentially related to the nature of the research, such as the study type (e.g., clinical, environmental, food safety) or the research approach (e.g., genetic, epidemiological).
  This plot reveals distinct clusters of keywords:
➢ Upper Right Quadrant: This area encompasses terms associated with genetic aspects and types of *E. coli* O157, including “stec” (shiga toxin-producing *E. coli*), “virulence genes”, and strain differentiations such as “O157” and “O157:H7”.
➢ Lower Right Quadrant: Terms in this section pertain to food sources and resistance, such as “beef”, “cattle”, and “antibiotic resistance”, highlighting a focus on the origins of *E. coli* O157 infections and the challenges in treating them.
➢ Left Quadrant: This portion includes broader terms like “spinach” and “lettuce”, linked to *E. coli* outbreaks, as well as general pathogens. It also encompasses terms like “*E. coli*” without specifying the O157 strain, indicating a more general research focus.
➢ Upper Left Quadrant: Here, other bacterial species like “*Staphylococcus aureus*” and “*Salmonella typhimurium*” are located, likely part of comparative studies in food safety or pathogenicity.

The relative distances between terms offer insights into the interconnectedness of research themes. For example, the central positioning of “*E. coli*” on the horizontal axis implies it is a primary focus, with research branching into specific areas like “O157”, “Shiga toxin”, and “foodborne pathogen”. This visualization proves invaluable for researchers, aiding their comprehension of the *E. coli* O157 research landscape. It helps identify closely related topics, potential research gaps, and emerging trends within the field.

## 5. Limitations

Database Limitation (Scopus): Our research exclusively relied on Scopus as the main database, owing to its extensive coverage of the scholarly literature across diverse fields. As Scopus is a reputable and widely acknowledged database, relying solely on it might result in overlooking pertinent studies available in other databases, potentially constraining the comprehensiveness of our analysis [100].

Language Restriction (English articles): We limited our analysis to English-language articles, introducing a potential language bias. However, by excluding non-English publications, we did not miss any valuable contributions from researchers in regions where English is not the primary language, as while using this filter, we ensured not to miss any potentially relevant studies on *E. coli* O157 research domain. Therefore, it can be said that this limitation has not led to a partial representation of the global research landscape on this topic [101].

Publication Bias: The analysis is vulnerable to publication bias since it primarily focuses on published articles. Published works often emphasize positive results, possibly neglecting studies with neutral or negative outcomes. This bias has not shown any potential to influence the overall interpretation of research patterns and themes [102].

## 6. Conclusions

The conducted bibliometric analysis has yielded insightful revelations within the domain of *E. coli* research, unraveling noteworthy patterns and advancements that delineate the historical evolution of investigations in this field. A pivotal milestone was the inaugural paper in the *E. coli* O157 research sphere in 1973, which triggered a notable upswing in publications commencing from 1993 onward. A striking observation is the pronounced influence of the United States in *E. coli* O157 research, as evidenced by a substantial output of 13,972 publications, surpassing other nations. This dominance extends to citations, totaling 105,195, and is accompanied by an impressive average article citation rate of 44.80.

Furthermore, the analysis highlights the “Journal of Food Protection” as the preeminent publication venue in this area, boasting 992 published articles and accruing a total of 41,238 citations. Eminent contributors to this domain include Li Y and Doyle Mp, with 102 and 84 publications, respectively, underscoring their significant contributions. Additionally, this study delves into the intellectual framework using techniques such as theme mapping, trend topic analysis, and multiple correspondence analysis (MCA) analysis. These methodologies shed light on emerging research themes and identify existing research gaps, thereby offering a comprehensive perspective for researchers, policymakers, and industries engaged in *E. coli* O157 research. Such insights hold considerable value for steering future research initiatives, shaping policies, and guiding industry practices within this critical domain.

## Figures and Tables

**Table 1:** Overview of traditional and novel antimicrobials used against *E. coli* O157.

Category	Antimicrobial Agent	Mechanism of Action	Effectiveness	Use in Treating *E. coli* O157	Notes on Resistance	References
Traditional Antimicrobials	Ciprofloxacin	Inhibits DNA gyrase	Highly effective	Used for severe cases; oral and IV forms	Emerging resistance observed	[32]
	Tetracycline	Protein synthesis inhibitor	Moderate effectiveness	Often used in veterinary settings	High levels of resistance reported	[33]
	Penicillin	Cell wall synthesis inhibitor	Limited effectiveness	Not typically used due to resistance	Widespread resistance	[34]
	Kanamycin	Aminoglycoside; inhibits protein synthesis	Effective against a broad range of bacteria	Used for serious infections; parenteral administration	Resistance can develop through enzymatic modification	[35]
	Cefoperazone	Third-generation cephalosporin; inhibits cell wall synthesis	Effective against Gram-negative bacteria	Used in serious bacterial infections	Resistance can occur via beta-lactamase production	[36]
	Streptomycin	Aminoglycoside; inhibits protein synthesis	Effective against a broad range of bacteria	Limited use due to resistance	High levels of resistance, especially in food animals	[37]
	Sulfisoxazole	Sulfonamide antibiotic; inhibits folate synthesis	Effective against Gram-negative bacteria	Limited use due to resistance	Resistance can develop through enzymatic pathways	[38]
New or Alternative Antimicrobials	Carvacrol (Food Additive)	Disrupts cell membrane	Potent antibacterial effects	Potential use as a natural food antimicrobial	Less prone to resistance development	[39]
	Sophorolipids	Disrupts cell membrane integrity	Greater activity than free-acid counterparts	Potential application in food and other industries	Emerging research, resistance not well characterized	[40]
	Bacteriophages	Target specific bacterial cells	Effective in reducing contamination	Used in the food industry and animal husbandry	Specificity reduces the chance of resistance	[41]
	Benzyl Isothiocyanate	Inhibits bacterial motility and toxin production	Effective against Salmonella and *E. coli* O157:H7	Potential for reducing infection in food products	Limited data on resistance	[42]
	Coumarins	Reduce biofilm formation and virulence	Effective antivirulence strategies	Potential for reducing persistent infections without affecting growth	Research in progress; resistance potential not fully explored	[43]
	Citrus-Based Natural Extracts	Natural antimicrobials; mechanism varies	Variable inhibitory activity against *E. coli* O157:H7	Could be used in pathogen-reduction strategies in foods	Natural origin may reduce resistance to development	[44]
	ε-Poly-L-Lysine	Disrupts membrane integrity, oxidative stress, and gene expression	Antibacterial against *E. coli* O157:H7	Potential use in food preservation and safety	Emerging research, resistance mechanisms not yet clear	[45]
	*Quercus infectoria* and *Punica granatum* Extracts	Natural antimicrobial agents; mechanism not fully understood	Show significant inhibition of *E. coli* O157:H7	Potential natural alternatives for reducing pathogens in food	Limited information on resistance development	[46]
	Probiotic Agents	Compete with pathogens for adhesion sites and nutrients	Mixed success in treating *E. coli* O157:H7 infections	Could reduce the risk of severe complications in infections	Generally low risk of developing resistance	[47]
	Plant-Derived Essential Oils	Various mechanisms, including membrane disruption	Variable effectiveness	Potential natural antimicrobials for food safety	Generally lower risk of resistance	[48]
	Silver Nanoparticles	Antimicrobial activity through multiple mechanisms	Effective against a wide range of microorganisms	Potential use in coatings and food packaging	Resistance mechanisms are less understood	[49]
	Lytic Enzymes	Degrade bacterial cell walls	Effective in targeting specific bacteria	Potential application in food safety and therapeutics	Specificity reduces the likelihood of resistance development	[50]
	Vanillin	Membrane depolarization and leakage of nucleic acids and proteins in the cell.	Highly Effective	Potential as a plant-derived antimicrobial.	Less resistance was reported.	[51]

**Table 2:** Recent detection technique for the diagnosis of *E. coli* O157 infection.

S No.	Technique	Type	Sensitivity	Reference
1.	Lateral flow immunoassay (LFIA) with silver enhancement	Immunoassay	Visual Limit of detection from 2 × 10^6^ (CFU mL^−1^) to 2 × 10^3^ (CFU mL^−1^)	[53]
2.	Paper-based fluorescent phage biosensor with smartphone detection	Biosensor	Detection limit as low as 50 CFU/mL	[54]
3.	Magneto-plasmonic nanosensor (MPnS) integrating SPR properties with T2 MR technology	Nanosensor	Detection as low as 10 CFUs	[55]
4.	Phage-based luminescence detection assay during enrichment while the sample is in transit	Bioluminescence	The potential of using the ΦV10nluc phage-based luminescence detection assay during enrichment	[56]
5.	Electrochemiluminescent (ECL) biosensor using NaBiF4 up-conversion nanoparticles	Biosensor	ECL behaviors of NaBiF4:Yb3+/Er3+ up-conversion nanoparticles for the detection of *E. coli* O157:H7	[57]
6.	Multiplex asymmetric PCR (MAPCR) with a chromogenic DNA microarray	PCR and DNA microarray technology.	The multiplex assay demonstrated outstanding sensitivity at 10 pg/μL and detection limits ranging from 10^4^ to 10^5^ CFU/25 g.	[58]
7.	rRAA assay integrated with TOMA	Biosensor	The limit of detection was less than 10 CFU mL^−1^	[59]
8.	Loop-Mediated Isothermal Amplification (LAMP)	PCR	The test demonstrated a sensitivity of 100%, a specificity of 97.05%, and an efficiency of 97.5%. Additionally, it showed a negative predicted value of 100% and a positive predicted value of 85.7%.	[60]
9.	Multivalent aptamer	Biosensor	Limit of detection of 10 cells per 250 mL	[61]
10.	Colorimetric/fluorescent dual-mode immunochromatographic assay	Immunochromatographic	The detection limit of 9.06 × 10^1^ CFU/mL	[62]
11.	Colorimetric sensor	Biosensor	The detection limit of 116 CFU/mL.	[63]
12.	Phage-apoferritin@CuO2 (phage-Apo@CP) probe	Biosensor	Limit of *detection* of 30 CFU/mL	[64]
13.	ELISA with streptavidin scaffolded DNA tetrads	ELISA	The fluorescence ELISA exhibited a detection limit of 3.75 × 10^3^ CFU/mL, which was 6.16 times superior to that of the conventional ELISA.	[63]
14.	PMA-PSR (propidium monoazide-polymerase spiral reaction)	Dye-based reaction	With a specificity rate of 100%, PSR had the capability to quantify DNA quantities as minimal as 1.12 pg/μL.	[65]
15.	PMA-mPCR	PCR	The mPCR assay demonstrated a detection limit of 10^3^ CFU/mL in the culture broth, while the PMA-mPCR assay exhibited a detection limit of 10^4^ CFU/mL in both pure culture samples.	[66]
16.	Ultrasensitive and specific Phage@DNAzyme signal probe	Combination of phages and DNAzymes	The detection limit was 50 CFU/mL	[67]
17.	Colorimetric Loop-Mediated Isothermal Amplification	Calorimetric	The cLAMP assay successfully identified 1 × 10^1^ CFU/g at a temperature of 65 °C for both genes, and its specificity for *E. coli* O157:H7 was confirmed.	[68]
18.	Nano enzyme-linked immunosorbent assay	ELISA	Detection sensitivity (8.7 × 10^2^ CFU/mL)	[69]
19.	RAA-CRISPR/Cas12a System	CRISPR-CAS	The method exhibited remarkable sensitivity, allowing the detection of as few as approximately 1 CFU/mL (using the fluorescence method) and 1 × 10^2^ CFU/mL (using the lateral flow assay) of *E. coli* O157:H7.	[70]
20.	Primer exchange reaction with glucose meter	Next-Generation Sequencing	The ability to identify *E. coli* O157:H7 with detection limits as minimal as 10 CFU/mL and within linear ranges of 10–10*7* CFU/mL was demonstrated.	[71]

**Table 3:** Most prolific author in the field of *E. coli* O157 research domain.

Author	H-Index	G-Index	M-Index	Total Citation	Total Number of Publication	First Publication Year
Doyle MP	49	84	1.256	9264	84	1985
Li Y	43	78	1.792	6240	102	2000
Beuchat LR	37	69	1.194	4868	77	1993
Karch H	35	47	0.946	3751	47	1987
Tarr PI	32	50	0.865	5073	50	1987
Besser TE	30	44	0.968	3709	44	1993
Kang D-H	30	48	1.2	2670	83	1999
Wang Y	28	55	1.077	3144	84	1998
Arthur TM	27	45	1.174	2492	45	2001
Bosilevac JM	27	45	1.286	2068	49	2003

**Table 4:** Most frequent author keyword in the research domain of *E. coli* O157 research.

S No.	Keyword	Occurrence	Total Link Strength
1.	*Escherichia coli* O157:H7	1065	2218
2.	*E coli* O157:H7	854	1771
3.	*Escherichia coli*	328	761
4.	*Salmonella*	298	926
5.	*Escherichia coli* O157	213	435
6.	*Listeria monocytogenes*	202	637
7.	*E. coli* 0157	197	429
8.	Food safety	189	457
9.	Foodborne Pathogens	184	414
10.	Cattle	159	461
11.	STEC	109	335
12.	Shiga Toxin	101	248
13.	Beef	95	280
14.	PCR	95	241
15.	Biofilm	91	214

**Table 5:** Most cited document in the research domain of *E. coli* O157 research.

S No.	Paper Title	Journal	Total Citation	Reference
1	“Genome sequence of enterohaemorrhagic *Escherichia coli* O157:H7”	Nature	2460	[86]
2	“Bifidobacteria can protect from enteropathogenic infection through production of acetate”	Nature	2331	[87]
3	“Complete Genome Sequence of Enterohemorrhagic *Escherichia coli* O157:H7 and Genomic Comparison with a Laboratory Strain K-12”	DNA Research	1551	[88]
4	“A genetic locus of enterocyte effacement conserved among diverse enterobacterial pathogens.”	PNAS	1581	[89]
5	“Epidemiology of *Escherichia coli* O157:H7 Outbreaks, United States, 1982–2002”	Emerging Infectious Disease	1735	[90]
6	“The Risk of the Haemolytic–Uremic Syndrome after Antibiotic Treatment of *Escherichia coli* O157:H7 Infections”	The New England Journal of Medicine	1511	[91]
7	“Pulse Net: The Molecular Subtyping Network for Foodborne Bacterial Disease Surveillance, United States”	Emerging Infectious Disease	1362	[92]
8	“An Outbreak of Diarrhea and Hemolytic Uremic Syndrome from *Escherichia coli* O157:H7 in Fresh-Pressed Apple Cider”	Jama Network	1220	[93]
9	“Quorum sensing in *Escherichia coli*, *Salmonella typhimurium*, and *Vibrio harveyi*: A new family of genes responsible for autoinducer production”	PNSA	1291	[94]
10	“Antibacterial activities of zinc oxide nanoparticles against *Escherichia coli* O157:H7”	Journal of Applied Microbiology	988	[95]

**Table 6:** Most Cited Journal in the research domain of *E. coli* O157.

Journal	H-Index	G-Index	M-Index	Total Citations	Total Number of Publications	First Publication Year
Journal of Food Protection	93	134	2.906	41,238	992	1992
Applied and Environmental Microbiology	91	143	2.333	25,604	323	1985
International Journal of Food Microbiology	80	119	2.424	20,869	379	1991
Food Microbiology	55	80	1.719	9776	264	1992
Biosensors and Bioelectronics	53	84	2.12	7234	96	1999
Journal of Clinical Microbiology	52	94	1.268	9072	101	1983
Journal of Food Science	52	81	1.733	8064	171	1994
Food Control	49	75	1.75	8460	252	1996
Journal of Applied Microbiology	49	87	1.815	8867	181	1997
Infection and Immunity	47	79	1.237	6354	80	1986

**Table 7:** Most cited countries.

Country	Total Citations	Average Article Citations
USA	105,195	44.80
Canada	17,814	40.30
China	17,803	24.60
United Kingdom	12,612	44.40
Korea	11,282	23.40
Georgia	11,027	57.40
Japan	9190	44.60
Spain	8491	49.90
Turkey	4922	25.20
Netherlands	3497	85.30

## Data Availability

Raw data are available from the corresponding author on request.
